# Pathways to peer interaction in ASD and TD through individual and dyadic joint-action motor abilities

**DOI:** 10.3389/fpsyg.2023.1234376

**Published:** 2023-09-18

**Authors:** Yael Estrugo, Shahar Bar Yehuda, Nirit Bauminger-Zviely

**Affiliations:** Faculty of Education, Bar-Ilan University, Ramat Gan, Israel

**Keywords:** motor functioning, joint action, peer interaction, autism, dyadic quality, social interaction

## Abstract

**Purpose:**

Any social engagement, especially with peers, requires children’s effective activation of social and motor mechanisms. Children and adolescents with autism spectrum disorder (ASD) often display dysfunctions both in individual motor functioning (e.g., fine/gross) and in dyadic joint action (JA), where two partners coordinate movement toward a shared goal. Yet, these mechanisms’ contribution to peer interaction has been underexplored.

**Method:**

This study examined the contribution of individual motor functioning and JA performance to peer interaction (cooperation, attentiveness, social engagement, and dyadic quality), while comparing children and adolescents’ (youngsters) with ASD versus those with typical development (TD).

**Results:**

Results indicated more competent peer interaction in TD than in ASD. Interestingly, only the ASD group showed significant maturation with age for social engagement and dyadic interaction quality, calls for further examination of developmental trajectories. However, even the oldest participants with ASD continued to lag behind the youngest TD group. Also, findings indicated that better individual motor functioning and JA performance explained better peer interactive competence; yet, the contribution of individual motor functioning to social cooperation and dyadic quality was moderated by JA performance. Thus, youngsters’ individual motor system was found to be an important contributor to peer interaction in those with low to moderate JA coordination capabilities, but not for those with high JA.

**Conclusion:**

Results emphasize possible distinct contributions of each motor mechanism and their interaction for facilitating social interaction, hence, encouraging incorporation of individual and dyadic motor skills explicitly into social interaction interventions for youngsters ASD.

## Introduction

Engagement of two partners or more in any social interaction – whether children are playing hide-and-seek, constructing a toy block model, or even walking side-by-side while talking – necessitates the activation of social and motor mechanisms via unconscious mimicry and body motions’ synchronization (Azaad et al., 2021). This link between the motor system and social interaction is well described conceptually by the “embodiment” theory, whereby humans’ physical bodies play a central role in shaping their experiences, understandings, and interactions in the world (Lux et al., 2021). Yet, for children and adolescents (youngsters) with autism spectrum disorder (ASD), who demonstrate well-documented peer interaction challenges and motor difficulties (Bhat, 2020, 2021; *DSM-5-TR*, American Psychiatric Association, 2022), the role played in peer interaction by motor functioning (within the individual) and socio-motor functioning (between individuals) has rarely been empirically examined in comparison to their counterparts with typical development (TD).

Across development, peers are an important source for cognitive, linguistic, and social growth (Hartup, 2009). Better understanding of the linkages between the body and peer-to-peer interaction during social engagement may lead to the development of customized interventions that facilitate those interactions both in ASD and TD. To narrow the gap in the literature, this study examined the links between individual and dyadic motor functioning with peer interaction, in both TD and ASD.

### Peer interaction in ASD

Autism spectrum disorder is a neurodevelopmental disorder characterized by difficulties in social communication and interaction; restricted, repetitive behaviors; and atypical exchange of sensory information (*DSM-5-TR*, American Psychiatric Association, 2022). Social interactions, mainly with peers, are regarded as core challenges for children with ASD (*DSM-V-TR*, American Psychiatric Association, 2022), often posing difficulties related to the production of complex interactive prosocial behaviors such as sharing, providing help, expressing positive affect, making eye contact, cooperating, and comforting (Bauminger-Zviely, 2013).

In general, the basic building blocks of adaptive peer interaction experiences develop differently in ASD, including the abilities to adjust one’s own behavior to suit a partner or a social context; maintain back-and-forth conversation; respond nonverbally to a partner’s communication (eye contact and facial expression); and integrate verbal with nonverbal communication modes such as looking towards and smiling at a peer while responding aloud to the peer’s overture (Bader and Fuchs, 2022).

The scarce empirical examination of peer interaction skills’ developmental trajectory with age in ASD suggest very little (Howlin et al., 2000) to no improvement across development in comparison with TD (Usher et al., 2015). More specifically, studies (e.g., Orsmond et al., 2004; Wallace et al., 2017) have found a decrease with age in social interaction and social-communication skills as measured using the well-accepted and parents-based Autism Diagnostic Interview – Revised (ADI-R – Lord et al., 1994) and Social Responsiveness Scale (SRS-2; Constantino and Gruber, 2012). Interestingly, Charman et al. (2017) have found that parent reported improvement in social functioning according to the SRS-2 between adolescence and adulthood in contradiction to adult’s self-report of a decline in social functioning in the SRS-2 between adolescence and adulthood. Those results highlight the need to further explore peer interaction development in ASD.

### Individual motor functioning in ASD

As far back as Rogers and Pennington (1991) highlighted motor difficulties as important in ASD symptomatology. Motor planning and motor coordination in individuals with ASD may be an important channel for understanding the social interaction deficit. Recent extensive reviews of motor functioning development from preschool to adulthood in ASD (Bhat, 2020, 2021; Licari et al., 2020; Zampella et al., 2021) have revealed substantial difficulties in: gross motor coordination (e.g., poor upper-limb and lower-limb coordination); locomotor skill coordination (e.g., running and jumping); fine motor coordination (e.g., grip planning, reaching, and grasping); gait and posture (e.g., shortened steps, toe walking, and balance); imitation and pantomime (e.g., of complex movement sequences); and motor planning, along with difficulties in organizing motor knowledge and longer reaction times during motion planning.

Motor difficulties in ASD can be observed in infancy (Provost et al., 2007; Brian et al., 2008), throughout childhood, and into adulthood (Ming et al., 2007), though irregularity decreases slightly with age (Fournier et al., 2010). Recent data from our autism research laboratory (Bauminger-Zviely et al., 2023) support this trend for less atypical motor functioning with increasing age: although children and adolescents with TD outperformed peers with ASD on gross and fine motor skills, in both study groups the older children (8.5–16 years) performed better than the younger children (6–8.5 years).

### Motor functioning’s linkages to peer interaction in TD and ASD

Despite the involvement of individual children’s motor skills in their peer engagement, not many studies yet have explored social-motor linkages (Cheung et al., 2021). Bar-Haim and Bart (2006) reported a connection between individual motor functioning and social play in kindergartners with TD during indoor and outdoor activities. Kindergartners with low motor abilities displayed less frequent social play and more social reticence compared to children with average or high motor abilities. Other ASD research (Hirata et al., 2014; Bhat, 2021; Fears et al., 2022) similarly found that larger motor impairments correlated with poorer social communication, social skills, and adaptive abilities, as assessed by several standardized diagnostic and evaluative measures of social-communication such as the Autism Diagnosis Observation Schedule (ADOS—2nd edition, Lord et al., 2012), the Social Communication Questionnaire (SCQ; Rutter et al., 2003), the SRS-2 (Constantino and Gruber, 2012), or the Vineland Adaptive Behavior Scales (Sparrow and Cicchetti, 1989). Importantly, better motor skills in infancy were correlated with lower social-communicative severity of ASD at older ages (e.g., Sutera et al., 2007), and the risk for motor impairment in ASD increases with higher social-communication deficits (Bhat et al., 2012; Bhat, 2021). Caregivers report of their autistic child’s motor and social performance also highlight the positive (e.g., practicing team playing) and negative (e.g., avoiding social-motor play due to clumsiness) effect social skills may have on the development of motor skills (Redquest et al., 2020). These findings imply that individual motor functioning may elucidate the social-communicative deficit in ASD and possibly may shed light on peer engagement in these children, calling for empirical investigation of social-motor linkages (Casartelli et al., 2016).

### Joint action (dyadic motor coordination) in TD and ASD

Since the individual’s (within-child) motor functioning is reciprocally connected with the peer dyad’s (between-children) motor functioning, the evaluation of the performance and practicing of dyadic motor coordination between peers can support the individual’s motor growth and development, and vice versa (Bowsher-Murray et al., 2022), calling for a parallel examination of children’s ability to coordinate their movements jointly with those of a partner.

Joint action (JA), where two partners coordinate movement toward a shared goal, is interactive by nature and enables mutually coordinated interaction in time and space (Sebanz et al., 2006). Almost any peer interaction involves JA, as when one child “mirrors” a friend’s leg movements during shared side-by-side walking or when children “complement” each other’s motions while playing a throw-and-catch ball game (Zampella et al., 2020). That is, JA comprises mirroring and/or complementing a partner’s body movements, based on the ability to predict another’s actions and their ramifications (Sebanz et al., 2006). JA findings, based on three recent reviews, report that JA capabilities are lower in ASD than in TD across a range of children’s ages and a variety of JA paradigms, mostly with an adult partner. These paradigms usually involve mirroring of hand movements, hand clapping, shared tapping or drumming, rhythmic movements, chair rocking, and body movements during a conversation, and complementing of hand movements in a moving target game (McNaughton and Redcay, 2020; Cerullo et al., 2021; Bowsher-Murray et al., 2022). Notably, the only two studies that implemented JA procedures with a peer partner (e.g., joint lifting of a bar with two hands and joint carrying of a table through a maze) of cognitively able (IQ > 75) children (6–12 years) and adolescents (8–18 years) with ASD presented specific JA difficulties compared to matched controls with TD (Stoit et al., 2011; Trevisan et al., 2021).

Regarding developmental trends for JA in social dyads, recent results from our autism research laboratory have shown lower JA capabilities with a peer partner in 84 ASD without Intellectual disability compared to 64 TD controls [matched according to IQ, chronological age (CA), and sex] across three developmental age groups: early childhood, preadolescence, and adolescence (Bar Yehuda and Bauminger-Zviely, 2022). Four JA mirroring and complementing tasks were executed in peer-dyads paired according to study group, IQ, CA, and sex. Though improvements in JA emerged towards adolescence, the adolescent ASD group’s JA performance level nonetheless resembled that shown by the TD group in early childhood (Bar Yehuda and Bauminger-Zviely, 2022). This JA procedure was examined in current study as one of the motor linkages with peer interaction (see method section for expansion on task’s procedure).

### JA linkages with peer interaction in TD and ASD

The ability to perceive and respond to social-communicative signals may have important consequences for individuals’ ability to adequately participate in movement coordination activities (Pezzulo et al., 2019), and thereby to develop adequate peer interaction skills (Bowsher-Murray et al., 2022). Research on children with TD has demonstrated that joint music making increases subsequent spontaneous cooperative and helpful behavior in 4-year-olds (Kirschner and Tomasello, 2010), and joint drumming correlated with social bonding in children ages 4–14 years (Howard et al., 2021). However, in ASD, the links between JA and peer interaction remain underexplored. In one rare study on ASD youngsters ages 11–16 years, Yoo and Kim (2018) reported significant improvement in social skills test scores following a dyadic joint rhythmic drumming intervention with an adult music therapist.

### The current study

Considering the potential importance of social-motor links for peer-to-peer engagement, this study’s main objective was to assess the contribution of children’s individual and dyadic motor functioning to their peer social interaction capability across development in participants with ASD compared to participants with TD. For peer interaction, we also examined age differences (early childhood, preadolescence, and adolescence) and group differences (ASD/TD) to elucidate developmental trajectories and identify possible vulnerable periods in ASD.

We hypothesized lower peer interaction abilities in the ASD group than the TD group, with no major changes along development (per Howlin et al., 2000; Orsmond et al., 2004; Usher et al., 2015). We also hypothesized that children with better individual and dyadic motor abilities would demonstrate better peer interaction capabilities (per Fitzpatrick et al., 2017; Bhat, 2021). Inasmuch as individual and dyadic motor capabilities have not been well studied simultaneously as predictors of social interaction, we could not hypothesize their relative contribution.

## Materials and methods

### Participants

Participants were 148 children and adolescents ages 6–16 years (118 males, 30 females) in two study groups: 84 with ASD without intellectual disability (IQ ≥ 70) and 64 with TD. Each study group spanned three developmental periods: early childhood (6–8.5 years), preadolescence (8.6–12 years), and adolescence (12.1–16.6 years). Inclusion criteria for the ASD group were: (a) score within the ASD range on the ADOS-2 (Lord et al., 2012), administered by the second author, and (b) IQ score >70 on the Wechsler (2010), administered by a clinical psychologist. Participants in the TD group were matched to the ASD group on CA, sex, mother’s education (to indicate socioeconomic status), and cognitive ability (IQ score). For the TD group, IQ score derived from two WISC-IV-HEB subtests, vocabulary (verbal) and matrices (perception), which reliably reflected cognitive ability in prior studies (Brezis et al., 2017; Trevisan et al., 2021) (Table 1).

**TABLE 1:** Participant characteristics and clinical phenotyping.

Background measures		ASD group (*n* = 84)			TD group (*n* = 64)			Statistical test
		Early childhood *n* = 22	Pre-adolescence *n* = 30	Adolescence *n* = 32	Early childhood *n* = 22	Pre-adolescence *n* = 20	Adolescence *n* = 22	
Chronological age (in months)	*M*	91.86	120.77	169.91	86.77	127.50	172.09	*F*(142) = 2.10
	SD	8.53	12.94	16.89	9.76	11.43	19.16	
Mother’s education^a^	*M*	5.18	4.72	5.22	5.68	5.65	5.82	*F*(141) = 1.22
	SD	1.14	1.25	1.31	0.57	1.14	0.73	
Sex	Male	20 (91%)	26 (87%)	24 (75%)	18 (82%)	16 (80%)	14 (64%)	*x*^2^(1) 1.56
	Female	2 (9%)	4 (13%)	8 (25%)	4 (18%)	4 (20%)	8 (36%)	
Cognitive ability^b^ (IQ)	*M*	102.27	109.17	100.94	117.95	108.25	116.82	*F*(142) = 1.41
	SD	28.15	32.27	32.46	30.54	20.67	15.24	
ASD severity (ADOS-2)	*M*	7.50	6.70	6.41				*F*_ASD_(81) = 3.70* Adolescence > early childhood
	SD	1.44	1.62	1.34				

ASD, autism spectrum disorder; TD, typical development; ADOS-2, Autism Diagnosis Observation Schedule—2nd edition.

^a^Mother’s education: 1, elementary; 2, high-school; 3, matriculation; 4, non-academic higher education; 5, BA; 6, MA; 7, Ph.D.

^b^IQ, mean score of vocabulary and matrices subtests. *p < 0.05.

### Measures

#### Observed peer interactions

Pairs of participants performed a joint Marbleworks© construction task in 42 ASD-ASD dyads and 32 TD-TD dyads (i.e., 74 fixed dyads). Dyads in each study group (ASD/TD) were matched, by sex, CA (<12 months between partners), and cognitive ability (<1 SD between partners). In addition, ADOS-2 difference between the group of partners 1 and partners 2 in the ASD dyads according to ANOVA, was not significant (ADOS-2 partner 1, *M* = 6.67, SD = 1.69; ADOS-2 partner 2, *M* = 6.93, SD = 1.33; *F* = 0.62, *p* > 0.05). Two measures of peer interactive behavior were assessed via a 10-min observation of each dyad’s videotaped shared construction game. In this scenario, children were provided with a noncompetitive construction game—Discovery Toys’ Super Marbleworks^®^ Raceway Construction Set. Children were instructed to construct a shared design (a marble maze-7 min) by using adjusted track pieces (i.e., ramps, connectors, funnels, and tunnels) to create pathways for dropping marbles down the track. Then, children roll the marbles down and through the maze (3-min). This procedure was found successful for differentiating social interaction behaviors in children with and without learning disabilities (Siperstein et al., 1997) and in children with ASD versus with TD (Bauminger et al., 2008). The Friendship Observation Scale (FOS; Bauminger et al., 2008) assessed each individual child’s social behaviors during dyadic play. The Dyadic Relationships Q-Set (DRQ; Park and Waters, 1989) assessed the quality of the dyadic interaction. Two autism experts coded the FOS and DRQ scores.

##### Each child’s social behavior

The FOS is an interactional coding system that provides minute-by-minute and global evaluations of each child’s interactive behavior according to the following categories: cooperation, attentiveness, and social engagement. *Cooperation* is a frequency scale comprising four indices: (a) task-oriented conversation (e.g., negotiating and requesting information); (b) gesture (e.g., nodding and shrugging); (c) joint attention (e.g., pointing and looking); and (d) gaze (at body and/or action). The 10-min observation time was segmented into 20-s sections, enabling the two coders to separately code any interactive behavior by each child using each of the four cooperation indices. *Attentiveness* is a global evaluation of each child’s attentiveness over the whole observed interaction period, referring to the child’s recognition of and responsiveness to the peer partner’s needs during the construction game, coded on a 3-point Likert scale ranging from “not attentive” (1) to “very attentive” (3). *Social engagement* is a global evaluation of each child’s play facilitation behaviors, such as initiations and suggestions, coded on a 5-point Likert scale ranging from “engaged in less than 20% of the interaction time” (1) to “engaged in 80% or above of the interaction time” (5).

The two raters first coded the three FOS categories for a randomly selected 25% of the dyads’ videotaped interactions. After inter-rater reliability achieved 92.88% mean agreement on all four FOS cooperation indices and 82.73% mean agreement level for the attentiveness and social engagement categories, each coder then proceeded to code half of the remaining 75% of the videos.

##### Dyad’s interaction quality

The 38-item DRQ scale (Park and Waters, 1989), developed to evaluate dyadic quality of peer interaction, assessed each interaction on five dyadic quality dimensions: positive social orientation (e.g., “partners express enjoyment when playing together”); cohesiveness (e.g., “when one partner moves away, the other moves in coordination”); harmony (e.g., “offers and suggestions guide dyadic play”); responsiveness (e.g., “partners endorse each other’s attitudes and activity preferences”); and coordinated play (e.g., “partners work together to produce more complex or organized play than either would engage in alone”). Utilizing a forced-choice Q-set format, the two coders sorted the 38 items into seven piles for each dyad, using a fixed 3-5-7-8-7-5-3 distribution, with the sort ranging from items that were behaviors least characteristic of the dyad (in Pile 1, scoring 1) to items that were behaviors most characteristic of the dyad (in Pile 7, scoring 7) during the construction game.

Due to the relatively subjective evaluative nature of the DRQ, all observations were coded simultaneously but separately by the two coders. Coders obtained an average of 90% agreement, and all disagreements were discussed until agreement was reached. In preliminary analysis, the total DRQ score (the mean of all five categories) showed high correlations with each of the five categories (ranging from *r* = 0.77 to 0.93); thus, the total DRQ score (Cronbach’s α = 0.88) was used in this study to reflect the interaction’s dyadic quality.

#### Observed JA tasks

The JA data collected for this study were part of a larger project examining differences between ASD and TD in JA performance and developmental trajectories (see, Bar Yehuda and Bauminger-Zviely, 2022). For the current study, the same 74 fixed dyads as above (in the observed peer interaction task) performed four JA tasks with their peer partner. Two *mirroring* tasks comprised *walking* side-by-side and *hand and body* imitation. Two *complementing* tasks comprised walking towards one another from two sides of an imaginary narrow *corridor* marked by two lines on the floor, and kicking-and-catching an imaginary *soccer* ball. The author and an expert in special education/ASD coded each participant’s observed movements, according to his/her ability to imitate, resonate, and mirror as well as to complement the partner’s movements. Specifically, each of the four tasks yielded a coordinated movement score for each participant that was calculated as the ratio between the coordinated movement’s duration (i.e., hand and body imitation and corridor) or frequency (i.e., walking and imaginary soccer) performed simultaneously by both participants, and the total number of movement’s duration or frequency that each participant performed.

The two raters coded a randomly selected subset of 35 dyads (47%) from both study groups. All disagreements were discussed until raters reached consensus. Interrater reliability (kappa) for the JA tasks were 0.85 for walking, 0.92 for hand and body, 0.82 for corridor, and 0.73 for soccer. We used the JA-total mean score in the current study due to its positive correlations with all four JA tasks (*r* = 0.69 for walking, 0.68 for hand and body, 0.69 for soccer, and 0.74 for corridor; *p* < 0.001). See Bar Yehuda and Bauminger-Zviely (2022) for more details on JA tasks and coding procedures.

#### Child’s individual motor functioning

The child’s individual gross and fine motor abilities were evaluated by using the Individual Motor Observation Scale (IMOS) (unpublished coding manual; Bauminger-Zviely et al., 2017) purposefully developed for the current study. The IMOS and its coding scale were built in consultation with professionals specializing in movement and occupational therapy, based on former movement scales and motor developmental milestones (e.g., Hattie and Edwards, 1987; Henderson, 1992; Payne and Isaacs, 2020). The IMOS motor tasks were successfully practiced in a pilot study on 10 children with TD (6–16 years).

The IMOS included eight motor tasks examining gross motor upper body (i.e., bouncing a ball, throwing a ball toward the wall and catching it); gross motor lower body (i.e., skipping, jumping on one leg, and heel-to-toe walking); and fine motor functioning (i.e., cutting a straight line with scissors, cutting a curved line with scissors, and nailing three nails in a row with a hammer). Children’s individual motor performance was videotaped and coded by the first author and an expert in special education/ASD (different than the above), according to movements’ proficiency and accuracy. For examples of coding, see the Table 2; the full observation’s coding can be obtained from the authors.

**TABLE 2:** Individual Motor Observation Scale (IMOS): sample gross and fine motor items and coding (Bauminger-Zviely et al., 2017).

Motor task	Category	Criteria	Coding			Score range
Gross motor – bouncing a ball, performed twice: once using right hand, once using left hand	Performance	With/without instruction/demonstration	2 = ball bounced 5 times after instruction	1 = ball bounced 5 times after demonstration	0 = ball bounced fewer than 5 times	0–2
	Proficiency and accuracy	Location		1 = ball bounced while standing in same place	0 = ball bounced from different places	0–1
		Height		1 = ball bounced up to shoulder level	0 = ball bounced above shoulder	0–1
		Differentiation		1 = body parts differentiated: chest and shoulders stay static while arm, forearm, and hand move	0 = no movement differentiation	0–1
		Continuity		1 = ball bounced continuously without stopping	0 = ball stopped after every bounce	0–1
	Total score range					0–6 for each hand 0–12 total bouncing
Fine motor^a^ – cutting a line with scissors, performed twice: once to cut a straight line, once a curved line	Proficiency and accuracy	Precision		1 = cutting is along line	0 = cutting diverges from line	0–1
		Differentiation		1 = body parts differentiated: hand moves from wrist	0 = no movement differentiation: arm / forearm / back moves	0–1
		Grip		1 = scissors held properly (thumb in upper hole, index finger in lower hole)	0 = scissors are held inappropriately	0–1
	Total score range					0–3 for straight line 0–3 for curved line 0–6 total cutting

^a^No instruction/demonstration for fine motor cutting task.

After inter-rater reliability reached 94% agreement for gross motor (upper and lower body) and 93% agreement for fine motor on 25% of the observations, one of the coders completed the remaining 75% of videos. Preliminary analysis revealed that the total motor score showed high correlations with its two components, gross motor (*r* = 0.98, *p* = 0.001) and fine motor (*r* = 0.75, *p* = 0.001); thus, the total IMOS score was used in current study (Cronbach’s α = 0.86).

### Procedure

This article was a part of a larger project examining socio-communication and motor links, including several additional measures that are outside the scope of the current paper. For this project, 212 children and adolescents (128 with ASD, 84 with TD) were initially recruited via advertisement of study objectives to parents, colleagues, advocating organizations, and in social media, after receiving approval from the faculty’s ethics committee. A subset of 64 was then excluded from the current study: those with IQ below 70 (*n* = 30 with ASD) and those with CA 12+ months over their potential partners (*n* = 14 with ASD, *n* = 20 with TD). After receiving written parental consent, two sessions were held at our autism research laboratory. In the first session, we evaluated participants’ ASD diagnosis (in the ASD group) and cognitive ability (in both groups). In the second, we carried out the peer interaction, IMOS, and JA tasks, in counterbalanced order.

### Data analyses

#### Age and group differences in peer interaction

To investigate children’s interactive behaviors enacted within a dyadic context (FOS), we used a two-level generalized linear mixed model (GLMM) approach (e.g., Snijders and Bosker, 1999). The GLMM integrates the dyadic effect, that is, repeatedly measures individuals within dyads (Fitzmaurice et al., 2009). We used the GLMM to determine main effects on the FOS of Group (ASD/TD), Age (early childhood/preadolescence/adolescence), and Group × Age interaction. Our analysis was computed using Mplus V.8.3 (Muthén and Muthén, 2018). To evaluate Group, Age, and Group × Age effects on the quality of the dyadic interaction (DRQ), we used analysis of variance (ANOVA). The source of the Group × Age interaction in all analyses was determined using post hoc pairwise comparisons adjusted by Bonferroni’s correction, subject to the *p* < 0.05 rejection criterion (Bonferroni, 1936).

#### Motor and social links

To examine the correlations between dyadic motor (JA), individual motor (IMOS), and peer interaction measures (FOS cooperation, attentiveness, and engagement and DRQ total score), we used a series of Pearson correlation tests.

#### Prediction of peer interaction

Our regression analyses to predict participants’ scores on the three FOS categories (cooperation, attentiveness, and engagement) utilized generalized estimating equations (GEE) that, like the GLMM, allowed us to integrate the two-level structure of the data, namely, individuals’ scores within a dyadic framework (Hardin and Hilbe, 2013). The GEE included the following predictors: study group (ASD/TD), dyadic motor functioning (JA), individual motor functioning (IMOS), and the JA × IMOS interaction. Then, Cramér’s phi was calculated to examine the effect size of each predictor (0–0.005 = no effect, 0.005–0.10 = low, 0.10–0.15 = moderate, 0.15–0.25 = strong, 0.25+ = very strong). To predict participants’ scores for the quality of the dyadic interaction (DRQ), we used hierarchical regression to test for the contribution of Group (Step 1), JA (Step 2), IMOS (Step 3), and JA × IMOS interactions (Step 4).

#### Moderated mediation to peer interaction

To further understand how the relations between individual and dyadic motor functioning capabilities may contribute to peer interaction, we employed SPSS PROCESS macro moderated mediation model 14 (Hayes, 2018). This procedure allows for examination of direct and indirect effects of the predictor × (study group) on the dependent variable Y (peer interaction: FOS cooperation and DRQ) through the mediation of IMOS, subject to varying levels of JA (e.g., Ahuja et al., 2022).

## Results

### Group and age differences in peer interaction

#### FOS: group and age main effects

Table 3 shows the GLMM empirical results for predicting the FOS categories of cooperation, attentiveness, and engagement. A significant main effect of group (ASD/TD) emerged consistently across the three categories, supporting our hypothesis of better peer interaction capabilities in children with TD compared to children with ASD, beyond age (see model effects and marginal means of TD versus ASD in the table). Likewise, the significant main effect of age showed consistent development on all three FOS categories with age, on average, beyond group. More specifically, in cooperation adolescent surpassed early childhood; and in attentive and engagement behaviors, adolescents surpassed preadolescents and early childhood; and preadolescents surpassed early childhood (see Table 3).

**TABLE 3:** Two-way GLMM dyadic modeling results, for group and age effects and their interactions for the FOS – child’s social-interactive behaviors.

FOS – social interactive behaviors			
Descriptive stats	Cooperation	Attentiveness	Social engagement
Mean	11.75	2.14	3.38
SD	4.41	0.85	1.49
Intra class correlation	0.71	0.39	0.87
GLMM results	Estimate (SE)	Estimate (SE)	Estimate (SE)
**Main effects**			
Group	−4.25***(0.79)	−0.87*** (0.12)	−2.08*** (0.21)
Preadolescents versus young	1.62 (0.98)	0.30* (0.15)	0.60* (0.27)
Adolescents versus young	2.85** (0.96)	0.63*** (0.15)	1.24*** (0.26)
**Marginal means – group**			
Typical development (TD)	17.05^b^ (1.28)	3.23^b^ (0.20)	5.74^b^ (0.35)
Autism spectrum disorders (ASD)	12.80^a^ (1.19)	2.36^a^ (0.18)	3.67^a^ (0.32)
**Marginal means – age**			
Young	8.33^a^ (0.81)	1.43^a^ (0.12)	1.83^a^ (0.22)
Preadolescents	9.94^ab^ (0.74)	1.73^b^ (0.11)	2.43^b^ (0.20)
Adolescents	11.18^b^ (0.72)	2.05^c^ (0.11)	3.06^c^ (0.20)
**Interaction effects**			
Group × preadolescents	2.99 (1.94)	0.39 (0.30)	1.26* (0.51)
Group × adolescents	1.89 (1.90)	0.24 (0.29)	1.14* (0.50)
**Marginal means – interaction**			
TD young	13.42 (0.99)	2.41 (0.15)	4.32^d^ (0.26)
TD preadolescents	13.41 (1.04)	2.50 (0.16)	4.25^d^ (0.27)
TD adolescents	15.31 (0.99)	2.91 (0.15)	4.96^d^ (0.26)
ASD young	7.49 (0.99)	1.32 (0.15)	1.41^a^ (0.26)
ASD preadolescents	10.47 (0.85)	1.80 (0.13)	2.60^b^ (0.22)
ASD adolescents	11.27 (0.82)	2.06 (0.13)	3.19^c^ (0.22)

N_children_ = 148, N_dyads_ = 74. Latin letters for age-group mean ranking, “a” for lowest, based on multiple-pairwise comparisons, with different letters denoting significant differences. Model fit indices for *Cooperation*: χ^2^ = 0.59, df = 2, *p* = 0.75, CFI = 1.00, TLI = 1.01, RMSEA < 0.001, SRMR_within_ = 0.000, SRMR_between_ = 0.022; for *Attentiveness*: χ^2^ = 0.58, df = 2, *p* = 0.75, CFI = 1.00, TLI = 1.06, RMSEA < 0.001, SRMR_within_ = 0.000, SRMR_between_ = 0.024; and for *Engagement*: χ^2^ = 0.59, df = 2, *p* = 0.75, CFI = 1.00, TLI = 1.05, RMSEA < 0.001, SRMR_within_ = 0.000, SRMR_between_ = 0.024. Levels of CFI, TLI > 0.09 denoted above acceptance threshold. *p < 0.05; **p < 0.01; ***p < 0.001. Bold values represent signify significant results.

#### FOS: group × age interaction effects

The statistical interaction effect of group by age was significant only for the global FOS category of social engagement, yielding age differences between the groups (ASD/TD). As seen in Table 3, the interactions of study group were significant with preadolescence (*b* = 1.26, *p* < 0.05) and with adolescence (*b* = 1.14, *p* < 0.05). The multiple pairwise comparison showed that, only in the ASD group, social engagement develops gradually with age (from a mean of 1.41 in early childhood to 2.60 in preadolescents to 3.19 in adolescents, “a” versus “b” versus “c”). To be noted, despite this improvement in peer engagement capabilities shown over development in ASD, the oldest group’s mean score (*M* = 3.19) indicated functioning below the mean of the youngest group with TD (*M* = 4.32).

#### DRQ: group, age, and group × age interaction effects

**FIGURE 1:**
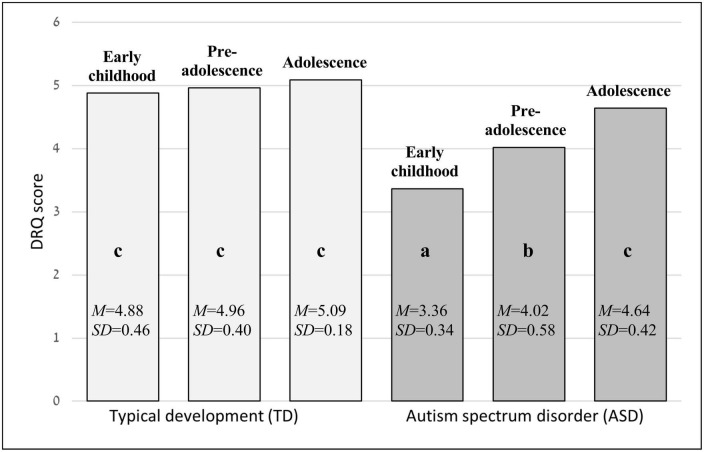
Group and age differences for quality of dyadic interaction (DRQ). Lowercase letters indicate significant intergroup differences where a < b < c; DRQ, Dyadic Relationships Q-Set.

The 2 (group) × 3 (age) ANOVA yielded a significant group effect, *F*(1,68) = 91.50, *p* < 0.001, *η_*p*_^2^* = 0.57, and a significant age effect, *F*(2,68) = 18.08, *p* < 0.001, *η_*p*_^2^* = 0.30. As seen in Figure 1, children with TD (*M* = 4.98, SD = 0.37) showed a significantly higher quality of dyadic peer interaction compared to children with ASD (*M* = 4.08, SD = 0.69). Quality of peer interaction improved with age, where preadolescents surpassed young children (*p* = 0.014), adolescents surpassed young children (*p* < 0.001), and adolescents surpassed preadolescents (*p* = 0.009). The Group × Age interaction effect was also significant, *F*(2,68), *p* < 0.001, *η_*p*_^2^* = 0.21, showing age improvement only in the ASD group, where preadolescents surpassed young children (*p* = 0.001), adolescents surpassed young children (*p* < 0.001), and adolescents surpassed preadolescents (*p* = 0.001). A further paired *t*-test executed between the youngest TD group and oldest ASD group revealed a nonsignificant difference, *t*(25) = −1.41, *p* = 0.591, indicating that adolescents with ASD showed performance similar to the youngest children with TD (see Figure 1).

### The role played by dyadic and individual motor functioning for peer interaction

#### Social-motor links

Pearson correlation tests were computed between peer interaction variables (FOS, DRQ) and motor functioning variables (JA, IMOS). Results yielded significant positive correlations for JA with all three FOS categories (cooperation-*r* = 0.26, *p* < 0.001; Attentiveness-*r* = 0.42, *p* < 0.001; social engagement-*r* = 0.45, *p* < 0.001) and with the total DRQ score (*r* = 0.56, *p* < 0.001), indicating that better coordinated JA was related to better peer interactive capabilities. Likewise, individual child motor functioning (IMOS) correlated positively with the three FOS categories (cooperation-*r* = 0.47, *p* < 0.001; Attentiveness-*r* = 0.48, *p* < 0.001; social engagement-*r* = 0.64, *p* < 0.001) and with the DRQ (*r* = 0.70, *p* < 0.001). In all cases, better individual motor skills were associated with better peer interaction capacities.

#### Prediction of peer interaction

As can be seen in Table 4, the GEE regression analyses yielded significant effects of Group, Dyadic Motor Functioning (JA), and Individual Motor Functioning (IMOS) for understanding all three peer interaction (FOS) categories, with strong to very strong effect sizes (Cramér’s phi). Overall, participants with TD showed higher FOS scores than ASD (Cooperation: effect = 3.38, *p* < 0.001, *ES* = 0.32; Attentiveness: effect = 0.52, *p* = 0.001, *ES* = 0.27; Social Engagement: effect = 1.50, *p* < 0.001, *ES* = 0.47). Also, better dyadic motor abilities (JA) significantly contributed to higher FOS scores (Cooperation: effect = 0.15, *p* = 0.025, *ES* = 0.18; Attentiveness: effect = 0.03, *p* = 0.006, *ES* = 0.23; Social Engagement: effect = 0.42, *p* = 0.002, *ES* = 0.26). Children’s better individual motor abilities (IMOS) also contributed significantly to higher FOS scores (Cooperation: effect = 0.38, *p* = 0.007, *ES* = 0.22; Attentiveness: effect = 0.06, *p* = 0.018, *ES* = 0.19; Social Engagement: effect = 0.08, *p* = 0.010, *ES* = 0.21). Taken altogether, children with higher motor capabilities showed higher levels of cooperation, attentiveness, and engagement in peer interaction, beyond group.

**TABLE 4:** Generalized estimating equations results for the contribution of study group, JA, IMOS, and JA × IMOS interactions to the explanation of peer interaction (FOS).

Predicted peer interaction (FOS) categories		Predictors				Model fit	
		Group (ASD/TD)	Dyadic motor coordination (JA)	Individual motor skills (IMOS)	JA × IMOS interaction	QIC	QICC
Cooperation	Wald χ^2^	**15.33**	**5.02**	**7.38**	**4.20**	2057.611	2055.353
	*p*	0.000	0.025	0.007	0.040		
	Effect (SE)	3.38 (0.86)	0.15 (0.06)	0.38 (0.14)	−0.01 (0.002)		
	95% CI (lower, upper)	(1.69, 5.08)	(0.02, 0.27)	(0.11, 0.65)	(−0.01, 0.000)		
	Cramér’s phi	0.32	0.18	0.22	0.17		
Attentiveness	Wald χ^2^	**10.94**	**7.50**	**5.56**	2.11	80.480	80.474
	*p*	0.001	0.006	0.018	0.146		
	Effect (SE)	0.52 (0.16)	0.03 (0.01)	0.06 (0.02)	−0.001 (0.0004)		
	95% CI (lower, upper)	(0.21, 0.83)	(0.01, 0.05)	(0.009, 0.10)	(−0.001, 0.000)		
	Cramér’s phi	0.27	0.23	0.19	0.12		
Social engagement	Wald χ^2^	**33.17**	**9.78**	**6.70**	2.62	163.345	160.841
	*p*	0.000	0.002	0.010	0.105		
	Effect (SE)	1.50 (0.26)	0.42 (0.01)	0.08 (0.03)	−0.001 (0.001)		
	95% CI (lower, upper)	(0.99, 2.04)	(0.02, 0.07)	(0.02, 0.14)	(−0.002, 0.000)		
	Cramér’s phi	0.47	0.26	0.21	0.13		

N_children_ = 148, N_dyads_ = 74. ASD, autism spectrum disorder; TD, typical development; JA, joint action; IMOS, Individual Motor Observation Scale; FOS, Friendship Observation Scale; CI, confidence interval; Cramér’s phi, effect size. *p < 0.05, **p < 0.01, ***p < 0.001. Bold values represent signify significant results.

Furthermore, for the FOS cooperation category, the interaction between dyadic (JA) and individual (IMOS) motor functioning contributed significantly to peer interaction, beyond their main effects (effect = −0.01, *p* = 0.040, *ES* = 0.17). The source of the statistical interaction was explored by using the PROCESS procedure (Model 1; Hayes et al., 2017) regardless of the data’s dyadic structure, yielding a significant effect of the IMOS on FOS cooperation for JA coordinated capabilities at 83.55% and less, based on the Johnson-Neyman test of continuous moderation. This finding indicates that the contribution of the child’s individual motor abilities to the explanation of cooperative peer interaction (FOS cooperation) decreases for participants with very high dyadic motor coordination (JA) capabilities.

Next, we examined the contributions of group, motor functioning (JA, IMOS), and JA × IMOS interactions to the explanation of the DRQ, using hierarchical regression. As seen in Table 5, the amount of variance (*R*^2^) explained by the combined independent variables and their interactions was 59%. In the first step, study group significantly contributed to DRQ (β = −0.62, Δ*R*^2^ = 38%, *p* < 0.001), with the TD group showing better quality of dyadic interaction than the ASD group. Next, the addition of JA in the second step contributed to the explained variance of DRQ (β = 0.35, Δ*R*^2^ = 10%, *p* = 0.001), with higher synchronization of dyadic motor functioning contributing to higher peer interaction quality. In the third step, the added individual motor functioning (IMOS) contributed an additional 7% to the explained variance in DRQ (β = 0.41, *p* = 0.001). Children with higher motor functioning had better peer interaction quality. Lastly, the IMOS × JA interaction was significant and added 3% to the explanation of the variance in DRQ (β = −1.34, *p* = 0.020). The source of this statistical interaction, examined using PROCESS, indicated that the effect of the IMOS on DRQ was significant and increased for JA coordination capabilities at 85.36% and less. This finding indicates that the contribution of the child’s individual motor abilities to the explanation of peer interaction (DRQ) decreases for participants with very high dyadic motor (JA) capabilities.

**TABLE 5:** Hierarchical regression results for the contribution of study group (ASD/TD), JA, IMOS, and JA × IMOS interactions to the explanation of dyadic quality of peer interaction (DRQ).

	Dyadic Relationships Q-Set (DRQ) (*n* = 78 dyads)		
Predictors	β	Δ *R*^2^	*p*
Step 1		**0.38**	**0.000**
Group	**−0.62**		0.000
Step 2		**0.10**	**0.001**
Group	**−0.46**		0.000
JA	**0.35**		0.001
Step 3		**0.08**	**0.001**
Group	**−0.26**		0.013
JA	**0.19**		0.055
IMOS	**0.41**		0.001
Step 4		**0.03**	**0.020**
Group	**−0.34**		0.002
JA	**0.88**		0.005
IMOS	**1.16**		0.001
IMOS × JA	**−1.34**		0.020
*R* ^2^		**0.59**	**0.000**

ASD, autism spectrum disorder; TD, typical development; JA, joint action; IMOS, Individual Motor Observation Scale. Bold values represent signify significant results.

#### Moderated mediation model for peer interaction

To further understand the unique contribution of motor functioning to peer interaction for those variables that yielded significant statistical interactions between individual and dyadic motor functioning in the regression model (i.e., FOS-Cooperation and DRQ-total), we examined a moderated mediation model. In this model, individual motor functioning (IMOS) mediated the link between group and peer interaction (FOS-cooperation and DRQ-total), while dyadic motor functioning (JA) moderated the path from IMOS to FOS, using Model 14 of the add-on PROCESS macro (Hayes et al., 2017).

##### FOS: individual child’s cooperation

**FIGURE 2:**
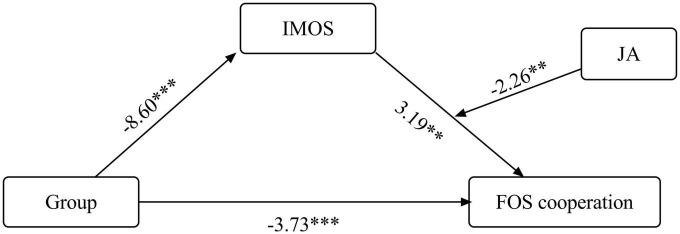
Direct and indirect relations between group (ASD/TD) and FOS cooperation via IMOS, moderated by JA. ASD, autism spectrum disorder; TD, typical development; FOS, Friendship Observation Scale; IMOS, Individual Motor Observation Scale; JA, joint action. *p < 0.05, **p < 0.01, and ***p < 0.001.

The index of the moderated mediation model was statistically significant (index = 0.06, 95% CI = 0.01, 0.06). Table 6 presents a detailed description of all stages of the analysis. Findings are summarized in Figure 2. As shown in Figure 2, study group was negatively related to IMOS, which in turn was positively related to FOS cooperation (where ASD = 1 and TD = 0). The significance of the mediation effect was estimated using a 95% CI, calculated based on bootstrapping of 5,000 samples. Because the value 0 was not included in the CI, the indirect relationship between group and FOS cooperation via IMOS was considered significant, but only for the first two levels (the mean and one SD below mean) and not for the third level (one SD above mean; see Table 6), as follows; the interaction in FOS cooperation (indirect relationship 95% CI for low level of JA: effect = −1.99, 95% CI 3.2320, −0.9166; CI for medium JA: effect = −1.34, 95% CI −2.5246, −0.2231; CI for high level of JA: effect = −0.53, 95% CI −1.9795, 0.9663). In other words, the ASD group showed lower IMOS scores, reflected in lower FOS cooperation scores.

**TABLE 6:** Indirect relationship between group (ASD/TD) and FOS cooperation via IMOS, moderated by JA: raw data for moderated mediation model.

	*B*	β	SE	95% CI	*t*	*p*
**IMOS predicted by group**						
Group	−9.35	−1.17	1.09	(−11.49, −7.20)	−8.60	0.000
**FOS cooperation as predicted variable**						
Group	−3.00	−0.68	0.80	(−4.59, −1.41)	−3.73	0.000
IMOS	0.52	0.26	0.16	(0.20, 0.84)	3.19	0.002
JA	0.17	−0.05	0.08	(0.007, 0.33)	2.06	0.042
IMOS × JA	−0.006	−0.15	0.003	(−0.01, −0.001)	−2.26	0.026
**Conditional indirect effects of the focal predictor at values of the moderator (JA)**						
Level 1: 1 SD < *M*	0.21	0.39	0.05	(0.11, 0.32)	3.94	0.000
Level 2: *M*	0.14	0.26	0.05	(0.04, 0.25)	2.76	0.007
Level 3: 1 SD > *M*	0.06	0.10	0.07	(−0.08, 0.20)	0.80	0.427

ASD, autism spectrum disorder; TD, typical development; FOS, Friendship Observation Scale; IMOS, Individual Motor Observation Scale; JA, joint action.

In addition, findings indicated that JA moderated the positive association between IMOS and FOS cooperation: the effect of the IMOS on FOS cooperation is significant for JA coordinated capabilities at 67% and less. As shown in Table 6, the conditional effects of IMOS on FOS cooperation at different levels of JA demonstrated that the positive relations between IMOS and FOS cooperation decrease as JA coordination level increases. This moderated the indirect link between group and FOS cooperation. The indirect conditional effects are presented at the bottom of Table 6 and on Figure 2.

##### DRQ: dyadic quality

**FIGURE 3:**
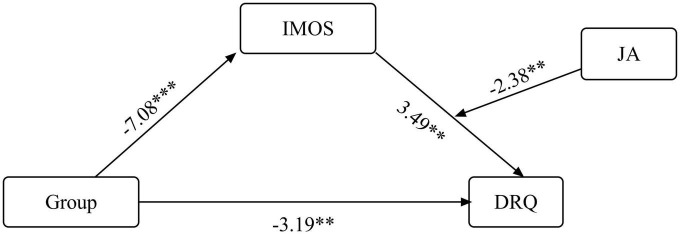
Direct and indirect relations between group (ASD/TD) and DRQ via IMOS, moderated by JA. ASD, autism spectrum disorder; TD, typical development; DRQ, Dyadic Relationships Q-Set. *p < 0.05, **p < 0.01, and ***p < 0.001.

The index of this moderated mediation model was also statistically significant (index = 0.01, 95% CI = 0.003, 0.01). Table 7 presents a detailed description of all stages of the analysis. As shown in Figure 3, study group was negatively related to IMOS, which in turn was positively related to DRQ. The significance of the mediation effect was estimated using a 95% CI, calculated based on bootstrapping of 5,000 samples. Because the value 0 was not included in the CI, the indirect relationship between group and DRQ via IMOS was significant, but only for the first two levels (the mean and one SD below mean) and not for the third level (one SD above mean; see Table 7), according to the interaction (indirect relationship 95% CI for low level of JA: effect = −0.46, 95% CI −0.7799, −0.2294; CI for medium JA: effect = −0.30, 95% CI −0.5659, −0.0760; CI for high level of JA: effect = −0.15, 95% CI −0.4625, 0.1551). In other words, the ASD group showed lower IMOS scores, reflected in lower DRQ scores.

**TABLE 7:** Indirect relationship between group (ASD/TD) and DRQ via IMOS, moderated by JA: raw data for moderated mediation model.

	*B*	β	SE	95% CI	*t*	*p*
**IMOS predicted by group**						
Group	−9.35	−1.28	1.32	(−11.98, −6.72)	−7.08	0.000
**DRQ as predicted variable**						
Group	−0.49	−0.67	0.15	(−0.79, −0.18)	−3.19	0.002
IMOS	0.11	0.34	0.03	(0.05, 0.18)	3.49	0.001
JA	0.05	0.16	0.02	(0.02, 0.08)	2.90	0.005
IOMS × JA	−0.001	−0.17	0.001	(−0.002, −0.0002)	−2.38	0.020
**Conditional indirect effects of the focal predictor at values of the moderator (JA)**						
Level 1: 1 SD < *M*	0.05	0.50	0.01	(0.03, 0.07)	4.26	0.000
Level 2: *M*	0.03	0.32	0.01	(0.01, 0.06)	2.76	0.007
Level 3: 1 SD > *M*	0.02	0.16	0.02	(−0.01, 0.05)	1.03	0.307

ASD, autism spectrum disorder; TD, typical development; DRQ, Dyadic Relationships Q-Set; IMOS, Individual Motor Observation Scale; JA, joint action.

In addition, findings indicated that JA moderated the positive association between IMOS and DRQ: The effect of the IMOS on DRQ is significant for JA capabilities at 68.22% and less, and not significant for JA higher than 68.22%. As shown in Table 7, the conditional effects of IMOS on DRQ, at different levels of JA, demonstrate that the positive relations between IMOS and DRQ decrease with increasing JA coordination. This moderation mediates the link between group and DRQ. The indirect conditional effects are presented at the bottom of Table 7 and in Figure 3.

## Discussion

The current study directed novel empirical scrutiny toward two mechanisms of motor functioning (individual and dyadic) simultaneously to help explain the observed abilities of children and adolescents with TD and ASD to cooperate, be attentive, and engage with their agemates, as well as the dyadic quality of such engagements. At first, we cross-sectionally examined the development of social-interactive skills from early childhood to adolescence in ASD versus TD. As expected, and in line with former studies looking at social interaction (Bauminger et al., 2003; Locke et al., 2016; Wallace et al., 2017), the TD group outperformed the ASD group – demonstrating better cooperative, attentive, and engaged behaviors in each peer interaction partner as well as higher levels of quality dyadic interaction.

Our findings regarding developmental trajectories of peer interaction were not as expected. Most informative are the significant group by age interactions that emerged for both the individual and the dyadic measures of peer interaction (FOS and DRQ), indicating growth along development only in the ASD group. The early childhood group demonstrated the lowest peer interactive capabilities, and the oldest adolescent group exhibited the highest, with preadolescents in between. Those findings showing social maturation in interactive capabilities in ASD differ from most prior studies that reported a plateau or regression in social interaction with age in this population (Howlin et al., 2000; Wallace et al., 2017; Franchini et al., 2018; Simpson et al., 2019; Scheeren et al., 2020).

Methodologies may partly explain this discrepancy between current and former results. We rated peers’ social interactive behaviors within a directly observed dyadic game situation, assessing each child’s cooperative verbal social behaviors (conversation, negotiations, and requests) and nonverbal ones (gestures, joint attention, and gazes). We also rated each observed child’s recognition of and responsiveness to their peer partner’s needs as well as their own social initiations and suggestions during the shared construction game. In contrast, previous studies utilized parent/caregiver questionnaires about children’s adaptive socialization and overall social competence (e.g., Wallace et al., 2017; Franchini et al., 2018), social interaction types (e.g., Scheeren et al., 2020), or social participation (e.g., Simpson et al., 2019). Another explanation of this discrepancy may involve participants’ ages because some studies examined adults’ outcomes (e.g., Howlin et al., 2000; Wallace et al., 2017), and others included outcomes for younger children using a large age range. For example, Franchini et al. (2018) studied preschoolers, but Simpson et al. (2019) and Scheeren et al. (2020) included children and adolescents.

Although variability in methodologies hampers comparison, we can carefully conclude from our results that improvement in interactive capabilities with age is conceivable in youngsters with ASD (without intellectual disability), referring to peer interactions occurring during the current semi-structured type of social game activity. Inasmuch as non-structured social settings like school recess would pose greater challenges for the social interactive skills of youngsters with ASD, future studies would do well to further explore our results using a wider variety of peer dyadic social experiences.

However, it is important to note that, notwithstanding the stability in group differences found along development for peer interactive skills, our GLMM comparison of all 6 groups (3 developmental ages × 2 study groups) indicated that even the oldest participants with ASD still performed significantly lower than the youngest participants with TD. Thus, our findings may have uniquely demonstrated the potential for significant developmental growth in social engagement capabilities in ASD; yet, these youngsters nonetheless do not close the gap with TD for peer interaction skills.

Following our cross-sectional and developmental analyses, we next aimed to shed light on the complex underlying motor mechanisms that may contribute to adaptive peer interactive skills in TD and ASD, we examined each child’s dual motor systems that are essential in everyday social interactions, and their statistical interactions: on the one hand the child’s own bodily motor skills and on the other hand the child’s ability to jointly coordinate body movements in space with those actions performed by a peer partner. As expected, the youngster’s individual motor system (IMOS) was linked with and predicted all peer interaction dimensions; namely, youngsters with better motor skills demonstrated better social interaction capabilities. This finding highlights the importance of individual motor skills for adaptive peer interaction, beyond the effects of group and age. Many social activities involving peers in classrooms or on the playground require gross motor capabilities such as running, chasing, hiding, and jumping as well as fine motor skills during various board games as well as their combination such as in a kick-and-catch ball game. Some motor requirements are more explicit in certain peer-to-peer activities; for example, gross motor skills are essential for a successful soccer game, whereas the importance of nonverbal movements during conversation is more implicit. Overall, either explicitly or implicitly, the child’s individual motor functioning is significant for peer interaction.

Our results for the contribution of motor mechanisms to peer interaction coincides with former studies that examined this link in TD children (Bar-Haim and Bart, 2006) and others that explored the link between the motor system and youngsters’ adaptive social communication skills in ASD (Hirata et al., 2014; Holloway et al., 2018; Bhat, 2021; Craig et al., 2021; Fears et al., 2022). Our findings extend the existing literature by demonstrating a link between the child’s motor system and peer-to-peer interaction during a real-world social play situation rather than with an adult experimenter or through various questionnaires, as found in most previous studies. All in all, our study findings suggest a motor channel that may facilitate peer interaction in ASD, supporting the inclusion of motor training into social intervention.

Youngsters’ own bodily motor capabilities and their joint motor coordination with a partner are two interlinked systems, yet not synonymous. Considering that JA incorporates the child’s own motor movements into the interaction with a peer partner’s motor movements, JA is a socio-cognitive-motor process. It integrates motor skills such as gross or fine motor planning or both, depending on the action goal at hand, as well as ample social and prosocial behaviors like sharing and joint attention, along with social-cognitive abilities like mentalization of others’ intentions or deciphering of their physical actions (Vesper et al., 2017; Cerullo et al., 2021; Emanuele et al., 2021; Howard et al., 2021). Thus, JA requires both partners to attribute and predict the beliefs, intentions, and emotions of each other in relation to the self (Fitzpatrick et al., 2018). It also requires accurate communication and interpretation of facial expressions, gestures, postures, and bodily maneuvers (Pezzulo et al., 2019).

Prior findings indicated that JA ability shapes many interactive activities (e.g., Howard et al., 2021). Our results confirmed its relevance to the context of peer-to-peer interaction. Youngsters with better ability to jointly coordinate their action with a partner (JA) demonstrated better FOS abilities – to collaborate with their peer partners and to attain higher levels of attentiveness and engagement – as well as a higher dyadic quality of interaction (DRQ). Notably, our study’s significant statistical interaction findings also underscore the unique predictive role played by the child’s dyadic motor system (JA) in contributing to peer interaction (FOS-cooperation and DRQ) beyond the contribution of the individual child’s own motor system alone (IMOS). Moreover, our moderated mediation model results revealed that JA moderated the individual motor functioning’s (IMOS) mediation of the link between study group and peer interaction (FOS-cooperation, DRQ). Thus, the youngster’s individual motor system was found to be an important contributor to peer interaction in those with low to moderate JA coordination capabilities, but not for those with high JA abilities. Our assumption is that, for youngsters with enhanced motor coordination abilities, other social-emotional-cognitive processes are more essential while the motor system may be less central. Previous research has shown that degree of coordination with a partner was significantly associated with children’s feelings of social closeness (Howard et al., 2021). Thus, perhaps dyads with higher joint coordination may focus on partners’ social-emotional togetherness and may be less concerned with related motor movements.

This dynamic between the child’s dyadic motor system and individual motor system as a path toward peer cooperation and dyadic interaction quality is intriguing and has theoretical as well as practical implications. In theory, through the examination of both motor mechanisms, we discerned the unique contribution of each to peer interaction and understood those abilities involvement in cooperative and productive social interactions construction. Practically, due to the challenges facing children and adolescents with ASD in both motor mechanisms, intervention planners should consider integrating both individual and dyadic motor skills to facilitate peer interaction.

Our study also holds several limitations. First, our observational method offers important benefits, but future researchers should combine it with other evaluation sources such as the Developmental Coordination Disorder Questionnaire (DCDQ; Wilson et al., 2009), which can provide parents’ perspective on their child’s individual motor functioning. Likewise, our IMOS instrument focused mainly on gross and fine motor skills, but other motor capabilities may be important to investigate for peer interaction, such as motor planning and motor control. The latter includes feedforward control (anticipating the social partner’s action) and feedback control (adjusting and timing one’s own action accordingly; Seidler et al., 2004).

In addition, our study utilized an ecologically valid peer interaction construction task that was efficient in differentiating between ASD and TD along development. Yet, this semi-structured setting may be less challenging for youngsters with ASD compared to more spontaneous non-structured free play situations, representing a real-time peer-to-peer activities commonly encountered in school. Furthermore, some former data point to the fact that there may be differences in peer interaction according to the type of the partner (ASD-ASD in non-mixed dyads versus ASD-TD in mixed dyads). Interactions with a TD peer seem to be important for the enhancement of complex social behaviors and for modeling of normative social interaction-but might be less reciprocal, whereas interaction with ASD partner may be more reciprocal and offer a sense of familiarity, and identification with someone like oneself, thereby holding importance for children’s self-esteem (see review in Bauminger-Zviely, 2013). Having said that, it will be interesting to further explore the socio-motor link also in mixed ASD-TD pairs.

Overall, our study results hold novel theoretical and therapeutic implications. Individual and dyadic motor mechanisms contributed significantly to youngsters’ ability to form cooperative interaction, to be attentive to partners, and to engage productively with their peers. Our findings suggest that the challenges experienced by youngsters with ASD in both motor mechanisms may provide an important explanation to one of the roots to the peer interaction deficit attributed to ASD (Cheung et al., 2022), calling for these mechanisms’ inclusion into social interventions. Integration of motor skill training and dyadic motor coordination activities into intervention may offer an important novel pathway to change, which may lead to a reduction in the loneliness and social isolation reported for school-age children with ASD alongside increases in their sense of social belonging and well-being (Bauminger-Zviely, 2013).

Moreover, the current outcomes furthered understanding of these two motor mechanisms’ interactions and distinct contributions to the facilitation of peer interaction, which may enable social interventionists to customize intervention goals more accurately. For example, the statistical interaction found between individual and dyadic motor functioning for FOS cooperation and for the DRQ suggests that high levels of joint socio-motor coordination (JA) may provide a compensatory mechanism enabling productive and successful peer interaction even in the face of poor individual motor capabilities (IMOS). Explicitly improving these youngsters’ JA skills – their ability to coordinate their physical actions with a peer partner – may be important to facilitate peer interaction despite individual motor difficulties (Bhat, 2021). Former studies indeed found that peer engagement in early childhood is an important index of adaptive functioning in the general population (e.g., Vaughn et al., 2016). Likewise, individual variations in peer interaction at early ages predicted later social competence (Rose-Krasnor and Denham, 2009). Future researchers and interventionists would thus do well to pursue improvement in peer interactive capabilities through both motor channels, to foster more socially adaptive functioning in children, preadolescents, and adolescents with ASD.

## Data availability statement

The datasets presented in this article are not readily available because our etic permission do not allow sharing participants raw data. Requests to access the datasets should be directed to NB-Z, nirit.bauminger@biu.ac.il.

## Ethics statement

The studies involving humans were approved by the Faculty of Education Ethics Committee. The studies were conducted in accordance with the local legislation and institutional requirements. Written informed consent for participation in this study was provided by the participants’ legal guardians/next of kin.

## Author contributions

YE and NB-Z contributed to the conception of the study. YE organized the data base and preformed the statistical analysis. All authors wrote sections of the manuscript and contributed to the design of the study, manuscript revision, read, and approved the submitted version.
